# Genome‐Wide 6mA Map Unveils Epigenetic Adaptation in Deep‐Sea Limpet

**DOI:** 10.1002/ece3.73449

**Published:** 2026-04-08

**Authors:** Chengqin Chen, Jianhui Liu, Ruhao Zhuang, Kuo Ni, Minxiao Wang, Yuli Li, Jing Wang, Shi Wang, Wentao Han, Lisui Bao

**Affiliations:** ^1^ Fang Zongxi Center for Marine Evo‐Devo, MOE Key Laboratory of Marine Genetics and Breeding & Shandong Key Laboratory of Marine Seed Industry Ocean University of China Qingdao China; ^2^ Key Laboratory of Marine Ecology and Environmental Sciences and Deep Sea Research Center, Institute of Oceanology Chinese Academy of Sciences Qingdao China; ^3^ Laboratory for Marine Biology and Biotechnology Qingdao Marine Science and Technology Center Qingdao China; ^4^ Key Laboratory of Tropical Aquatic Germplasm of Hainan Province, Sanya Oceanographic Institution Ocean University of China Sanya China; ^5^ Southern Marine Science and Engineering Guangdong Laboratory Guangzhou China; ^6^ Institute of Evolution & Marine Biodiversity Ocean University of China Qingdao China; ^7^ SANYA Oceanographic Laboratory Sanya China

**Keywords:** *Bathyacmaea lactea*, deep‐sea cold seeps, DNA N6‐methyladenine (6mA), epigenetic adaptations, PacBio SMRT sequencing

## Abstract

How animals thrive in extreme deep‐sea environments remains a key question in marine evolutionary biology. While adaptation is often studied through the lens of genetic sequence, the epigenetic mechanisms that allow for phenotypic plasticity and rapid environmental response in these organisms remain a major “black box”. DNA N6‐methyladenine (6mA) is an emerging epigenetic mark in eukaryotes, but its presence and role in deep‐sea animals remain unknown. Here, we present the first genome‐wide map of DNA 6mA in a deep‐sea animal, the cold seep limpet *Bathyacmaea lactea*, generated using PacBio single‐molecule real‐time (SMRT) sequencing. We identified 281,772 high‐confidence 6mA sites, comprising ~0.13% of all adenines in the genome. Over 60% of genes harbor 6mA, with sites significantly enriched in exons. Integration with RNA‐seq data revealed that gene body 6mA correlates with active transcription, whereas promoter 6mA is associated with gene repression. Genes with abundant 6mA are enriched in pathways related to energy metabolism, protein homeostasis, and osmoregulation, suggesting that 6mA methylation may facilitate adaptation to high pressure, low temperature, and dark deep‐sea conditions. Our findings uncover 6mA as a component of the *B. lactea* epigenome and provide novel insights into epigenetic regulation under extreme deep‐sea environments.

## Graphical abstract

Our study provides the first genome‐wide 6mA profile for any deep‐sea animal, uncovers its sophisticated, context‐dependent role in gene regulation, and functionally links this epigenetic mark to the adaptive pathways essential for deep‐sea survival. This work paves a way to study deep‐sea 6mA epigenetics and provides a novel mechanism for understanding its adaptation to extreme environments.

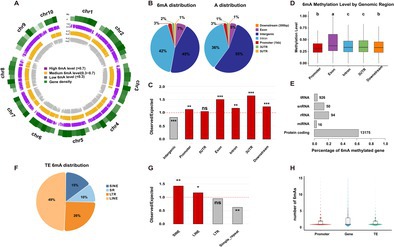

## 1 Introduction

Deep‐sea hydrothermal vents and cold seeps constitute oases of life on the seafloor and are home to unique life forms (Le et al. 2016; Brazelton 2017). Cold seeps are specialized marine environments primarily located along continental slopes and subduction zones, where methane, hydrogen sulfide, and other hydrocarbon‐rich fluids seep out (e.g., CH_4_, H_2_S) (Orcutt et al. 2011; Le et al. 2016; Niu et al. 2017; Vigneron et al. 2019; Dong et al. 2020). How deep‐sea animals have adapted to these unique environmental conditions remains an intriguing scientific question that is not yet fully understood. The deep‐sea limpet *Bathyacmaea lactea* is a common gastropod species associated with cold seep environments, often found in proximity to dominant mussel beds in chemosynthesis‐based communities (Zhang et al. 2016; Liu et al. 2020). As an endemic species in these habitats, it provides an important ecological component of seep benthic assemblages and serves as a promising model for studying adaptation to cold seep ecosystems (Chen et al. 2025). Recent genome sequencing has established this species as a promising model for investigating adaptive evolution in extreme deep‐sea environments (Chen et al. 2025).

DNA methylation is an essential epigenetic mechanism that responds to environmental stimuli and can facilitate adaptive phenotypic changes (Martin and Fry 2018; Wilkinson et al. 2021; Poggiali et al. 2024). It plays an important role in regulating genomic imprinting, transposon suppression, gene expression and a variety of cellular processes (Bergman and Cedar 2013; Liang et al. 2018; Kan et al. 2022; Mattei et al. 2022). Depending on the nucleotide being modified, the major forms of DNA methylation are 5‐methylcytosine (5mC), N6‐methyladenine (6mA), and N4‐methylcytosine (4mC) (Cheng 1995; Ratel et al. 2006; Lucas and Novoa 2023). 5mC is the most extensively studied epigenetic mark in eukaryotes (Vanyushin et al. 1970). Recent studies have also revealed the adaptive epigenetic role of 5mC in three deep‐sea species (*Paraescarpia echinospica, Ridgeia piscesae*, and *Paralvinella palmiformis*) (Perez et al. 2023). In contrast, 6mA methylation was long thought to be a hallmark of prokaryotic and protist genomes. However, several recent studies have now reported the presence of 6mA in eukaryotes, including ciliates (Zhao et al. 2023), algae (Fu et al. 2015; Gong et al. 2024; Liu et al. 2024), plants (Liang et al. 2018; Zhang et al. 2018; Zhou et al. 2018; Xie et al. 2020), invertebrates (Greer et al. 2015; Wang et al. 2018; Starczak et al. 2022) and vertebrates (Luo et al. 2015; Liu et al. 2016; Yao et al. 2017; Xiao et al. 2018; Boulias and Greer 2022).

6mA exhibits different distribution and diverse function across different eukaryotic species; several studies suggest that 6mA has a regulatory and responsive role to stress in algae, plants, animals, and humans (Koh et al. 2018; Beh et al. 2019; Wang et al. 2019; Boulias and Greer 2022). In *Chlamydomonas reinhardtii* , 6mA is enriched around transcription start sites and marks actively transcribed genes (Fu et al. 2015; Xiao et al. 2018). By contrast, in *Drosophila melanogaster* , 6mA affects the expression of certain transposons (Zhang et al. 2015). In *Arabidopsis thaliana* and humans, 6mA is enriched in exons of protein‐coding genes and shows a positive correlation with gene expression (Liang et al. 2018). In mouse brain, elevation of 6mA levels upon environmental stress upregulates the expression of neuronal genes and represses LINE transposons (Yao et al. 2017). Nonetheless, no comprehensive study has examined DNA 6mA in any deep‐sea animal species, so it remains unknown whether 6mA exists in their genomes or what role it might play in gene regulation and epigenetic adaptation to the deep‐sea environments.

Long‐read sequencing technology has been used to directly detect 6mA in multiple species, including *Escherichia coli* (Fang et al. 2012), *Nannochloropsis oceanica* (Gong et al. 2024), *A. thaliana* (Liang et al. 2018), and mouse (Wu et al. 2016). Single‐molecule, real‐time (SMRT) sequencing can identify epigenetic modifications at base‐pair resolution. It also provides improved read alignment quality and more mappable regions because it does not require base conversion (Flusberg et al. 2010; Lucas and Novoa 2023; Fu et al. 2025). Here we used PacBio SMRT sequencing technology to profile 6mA modifications in *B. lactea* , a lophotrochozoan gastropod that inhabits deep‐sea hydrothermal vents and cold seeps. In this study, we demonstrate for the first time that 6mA is present in the genome of the deep‐sea lophotrochozoan *B. lactea* . We found that 6mA modifications are significantly enriched in exons of protein‐coding genes and associated with active transcription. In addition, Gene Ontology (GO) analysis revealed that the highly 6mA‐methylated genes were enriched in homeostatic responses, osmotic equilibrium combined with metabolic adjustments. This study provides new insights into the epigenetic basis of deep‐sea adaptation in *B. lactea* .

## 2 Materials and Methods

### 2.1 Identification of 6mA in the *B. lactea* Genome

*B. lactea* specimens were collected in the South China Sea (22.10° N, 119.28° E; 1168 m depth) using a remotely operated vehicle (ROV) aboard the research vessel Kexue, as reported in the original publications (Chen et al. 2025). Raw PacBio sequencing reads were retrieved from the NCBI Sequence Read Archive (SRA) under the BioProject accession number PRJNA1237989. PacBio SMRT link (version 7.0.1) was used to detect DNA 6mA modification in the *B. lactea* genome (https://anaconda.org/HCC/smrtlink‐tools). Briefly, each raw dataset was aligned to the *B. lactea* reference genome using pbalign with default parameters. Alignment files were merged with Samtools v1.9. Lastly, 6mA sites were identified using ipdSummary.py script in SMRT link. DNA 6mA sites were identified from SMRT sequencing data based on polymerase kinetics, specifically inter‐pulse duration (IPD) signals aggregated across aligned subreads at each genomic coordinate. To obtain high‐confidence modification sites, positions with sequencing coverage lower than 30× were excluded from downstream analyses.

### 2.2 Computational Analysis of 6mA Density and Gene Expression

Genome‐wide 6mA distribution profiles were generated using circlize v0.4.17 (Gu et al. 2014). Based on the genome annotation (GFF3 file), genomic features were classified into promoter, gene body, downstream, and intergenic regions. Promoter regions were initially defined as 1 kb upstream of the transcription start site (TSS), and downstream regions were defined as 300 bp downstream of the transcription termination site (TES). Within gene bodies, regions were further classified into exons, introns, and 5′/3′ UTRs. In addition, repetitive elements in the genome were identified using RepeatMasker v4.1.2 with default settings (Smit et al. 2015). As modification detection in SMRT Link relies on integrated kinetic evidence across multiple aligned subreads rather than individual read counts, no additional MAPQ‐based filtering was applied at the read level. The overlap between 6mA sites and gene structures was determined using BEDTools v2.30.0 (Quinlan and Hall 2010), and the distribution of 6mA sites across different classes was subsequently calculated. To assess the robustness of our conclusions to promoter definition, we additionally performed the same enrichment analyses using alternative promoter windows of 500 bp and 2 kb upstream of the TSS. For the enrichment of 6mA in genomic features, we used adenine content as the background (null model), given that 6mA modifications occur exclusively on adenine bases. Expected values were calculated as the proportion of all genomic adenines located within each feature multiplied by the total number of detected 6mA sites. Enrichment was defined as the ratio of observed to expected 6mA counts. The significant difference of the observed and expected number of methylation sites was determined using the Fisher's exact test.

Short‐read RNA‐seq raw data from *B. lactea* muscle tissue were acquired from NCBI (accession number PRJNA1237989). The RNA‐seq reads were mapped against the *B. lactea* reference genome using STAR program with default parameters (Dobin et al. 2013). Gene expression levels were quantified as transcripts per million reads (TPM) (Wagner et al. 2012). To examine the relationship between gene expression and 6mA density, we grouped genes by quartiles of 6mA site occupancy Q1–Q4, from lowest to highest. Differences in expression between groups were assessed with Student's *t*‐tests.

### 2.3 Protein‐Coding Gene Annotation

Protein‐coding gene models and corresponding protein sequences were obtained from previously published genome annotations (Chen et al. 2025). To investigate the function context of 6mA methylation, we performed protein functional annotation by aligning protein sequences to the NCBI non‐redundant (nr) database (downloaded on March 5, 2025) and SwissProt database (release 2024_06) using BLASTP implemented in BLAST+ v2.12.0 (Camacho et al. 2009) with an *E*‐value cutoff of 1e−5. Protein domains were identified by searching against the Pfam database v32.0 using hmmsearch implemented in HMMER v3.3.2 (Eddy 2011) with an *E*‐value threshold of 1e−5 to define significant domain hits.

### 2.4 Motif Analysis and GO Analysis

For each 6mA site, we extracted the 4 bp flanking sequence on each side (9 bp total centered on the 6mA). Conserved motifs in the flanking regions of 6mA sites were detected using DREME implemented in MEME v5.5.0 (Bailey et al. 2009, 2015) with parameters ‘‐dna‐mink 4‐maxk 8’. The corresponding *E*‐values of the motifs were calculated using Fisher's exact test.

To reduce transcriptional noise, GO enrichment analysis was initially performed on genes with expression levels above the genome‐wide average TPM. This analysis was intended to characterize functional categories associated with actively transcribed genes rather than to exclude potential regulatory roles of 6mA in low‐expressed or repressed genes. All protein‐coding genes were classified into hypermethylated‐highly expressed (top 10% gene‐body 6mA level; TPM above average) and hypomethylated‐highly expressed (bottom 10% gene body 6mA level; TPM above average) groups. GO annotations were obtained using InterProScan, and GO enrichment analysis was performed using the clusterProfiler R package v4.6.2 (Wu et al. 2021) with ggplot2 v3.4.2 (Wickham 2016) for visualization. GO enrichment analysis was performed using a hypergeometric test, and *p*‐values were adjusted for multiple testing using the Benjamini–Hochberg false discovery rate (FDR) correction. GO terms with an adjusted *p*‐value (FDR) < 0.05 were considered to be significantly enriched. A chord plot was generated using the GOplot R package v1.0.2 (Walter et al. 2015). We additionally examined genes with all expression levels and did not observe strong GO enrichment signals, likely due to limited statistical power.

### 2.5 Multiple Sequence Alignments and Phylogenetic Analysis

To identify putative 6mA methylation enzymes, previously identified families of 6mA methyltransferase (DAMT‐1) and demethylase (NMAD‐1) were used as seed in integrative profile searches against a local protein database. Iterative sequence profile searches were done using PSI‐BLAST (Camacho et al. 2009) (*E*‐value ≤ 1e−5; minimum amino acid identity of ≥ 25%; a minimum query coverage of ≥ 60%) and JACKHMMER (Eddy 2011) (*E*‐value ≤ 1e−5). All candidate sequences identified by either approach were further validated by Pfam domain annotation, and questionable hits were manually inspected and curated to ensure domain architecture consistency with known DAMT‐1 and NMAD‐1 proteins. Homologous genes were identified in *B. lactea* and across 17 other species, including eight lophotrochozoans (*Argopecten purpuratus*, *Aplysia californica* , *Bathymodiolus platifrons*, *Elysia chlorotica* , *Lottia gigantea* , *Modiolus philippinarum*, *Patinopecten yessoensis*, *Pinctada fucata martensii*), four ecdysozoans ( *Caenorhabditis elegans* , *Daphnia pulex* , *Litopenaeus vannamei* , *Tribolium castaneum* ), three deuterostomes ( *Branchiostoma floridae* , *Danio rerio* , *Homo sapiens* ), and two Viridiplantae species ( *Arabidopsis thaliana* , *Oryza sativa* ). Multiple protein sequence alignments were performed using MUSCLE in MEGA 7.0 (Kumar et al. 2016). For the DAMT1 and NAMD1 datasets, the maximum likelihood (ML) phylogenetic trees were constructed with best substitution models LG + G + I + F and WAG + G + F found by ModelTest‐NG v0.1.7 (Darriba et al. 2020), respectively. Bootstrap analysis with 1000 bootstrap replicates was performed to obtain the confidence support level. A Bayesian Inference analysis was conducted using BEAST2 v2.6.7 (Bouckaert et al. 2014). The analysis was run for 100 million generations, with sampling every 100,000th generation, using the same substitution models selected for the ML analysis. We used a Yule Model as the prior. The first 10,000 samples were discarded as burn‐in to ensure convergence. Alignments were visualized using Jalview (Waterhouse et al. 2009). All sequences used in the phylogenetic analysis and their corresponding accession numbers are listed (Table S1).

## 3 Results

### 3.1 DNA 6mA Modification Occurs in the *B. lactea* Genome

**FIGURE 1:**
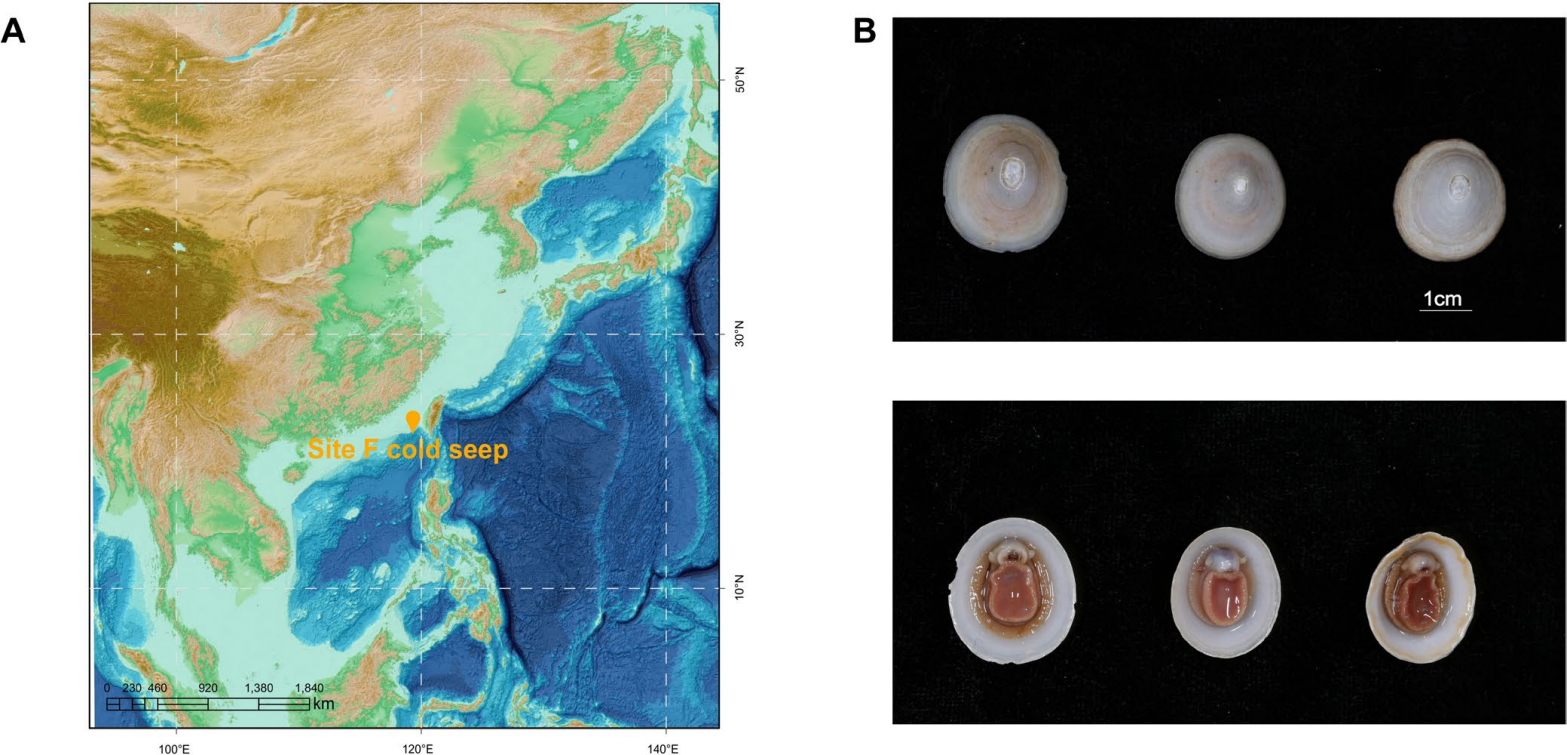
Images of *Bathyacmaea lactea*. (A) Study sampling sites in Site F Cold Seep (22.10° N, 119.28° E; 1168 m depth). (B) Frontal and dorsal views of three *Bathyacmaea lactea* individuals.

An overview of *B. lactea* and the geographic locations of the analyzed samples was shown in Figure 1. To characterize genome‐wide DNA methylation patterns, we analyzed SMRT sequencing data from the *B. lactea* genome and detected a total of 281,772 high‐confidence DNA 6mA sites (Table S2). To reduce noise interference from raw SMRT signals, we applied a two‐level filtering strategy for 6mA identification. Only SMRT sequencing datasets with a genome‐wide average coverage exceeding 100× were used to ensure sufficient signal quality. Within these datasets, high‐confidence DNA 6mA sites were further filtered based on site‐specific criteria, including Identification QV ≥ 20, a minimum per‐site coverage of 30×, and an IPD ratio ≥ 1.5, before downstream analyses (Flusberg et al. 2010; PacBio SMRT Link documentation). The overall 6mA density (6mA/A) in *B. lactea* was approximately 0.13%. Comparative analysis with other eukaryotic species showed that this level falls within the intermediate range of reported 6mA densities, being comparable to some plant and vertebrate systems, lower than those reported in several unicellular eukaryotes, and higher than those observed in certain multicellular animals and plants (Figure S1).

**FIGURE 2:**
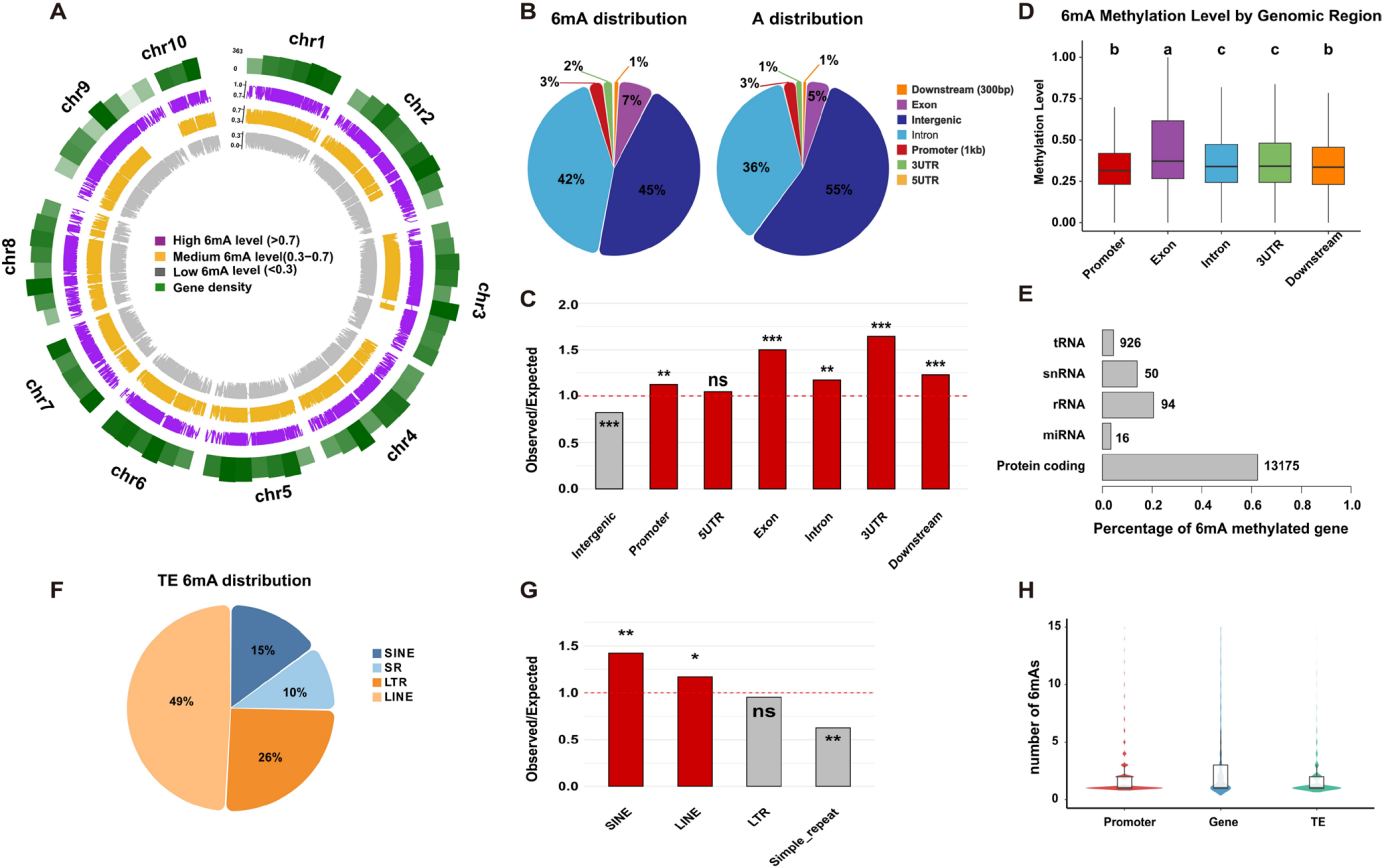
Distribution and global features of 6mA sites in *B. lactea* genome. (A) Circos plots of the density distribution of 6mA across all chromosomes in the different 6mA modification level. Dark purple, orange and gray circles represent highly methylated (0.7–1), moderately methylated (0.3–0.7) and lowly methylated (0–0.3) 6mA, respectively. (B) Pie plots show proportions of all 6mA sites and A sites in 5′ UTR, Promoter, 3′ UTR, Exon, Intron, Downstream and Intergenic regions. (C) Comparison of observed versus expected distributions of 6mA sites in each functional region. (D) Box plot of 6mA methylation level in significantly enriched functional regions. (E) Histogram shows percentages of genes containing 6mA modification in different gene categories. The number of 6mA‐modified genes are shown on the right. (F) Pie plots show proportions of all 6mA sites in SINE, Simple repeat (SR), LTR and LINE. (G) Comparison of observed versus expected distributions of 6mA sites in each transposable element. (H) Number of 6mA sites located on promoter regions, genes and TEs. ns, not significant; *p < 0.05; **p < 0.01; ***p < 0.001.

We divided the 6mA modification level into low (≤ 0.3, gray circle; Figure 2A), medium (0.3–0.7, orange circle), and high (≥ 0.7, dark purple circle) categories. The 6mA density was predominantly in the medium range, and regions with higher gene density tended to show higher 6mA density. To investigate the genomic distribution of 6mA sites, the genome was divided into promoter (defined as 2 kb, 1 kb and 500 bp upstream of the transcription start site), gene body, downstream, and intergenic regions (Figures 2B and S2). Gene bodies were further subdivided into exons, introns, and 5′/3′ UTRs. Nearly half of all 6mA sites were located within gene bodies, with 42% of 6mA sites residing in introns (Figure 2B). Notably, 6mA sites were significantly enriched in exonic regions relative to expectations (Figure 2C). 6mA sites were also enriched in promoters, exons, introns, 3′ UTRs and downstream regions, suggesting that 6mA may be involved in the regulation of gene expression (Figure 2C). Consistently, 6mA sites displayed higher methylation levels in exonic regions than in other parts of the gene (Figures 2D and S3). In addition, over 60% of the protein‐coding genes (13,175 genes) in the *B. lactea* genome had at least one 6mA site (Figure 2E), whereas only a small fraction of non‐coding genes was methylated (Figure 2E). To further characterize 6mA sites preference among repetitive elements, we examined major transposable element classes and observed significant enrichment of 6mA in LINE and SINE retrotransposons (comprising ~49% and 15% of all 6mA sites, respectively; Figure 2F,G). This suggests a preferential targeting of these elements by the 6mA methylation machinery. Furthermore, protein‐coding gene regions contained more 6mA sites than promoters or transposable elements (Figure 2H). Similar trends were observed when promoter regions were defined as 500 bp or 2 kb upstream of the TSS, supporting the robustness of the observed association between 6mA distribution and gene regulatory regions (Figure S2).

We next examined whether 6mA sites share consensus motifs in the *B. lactea* genome. The most enriched motifs sequences at 6mA sites in *B. lactea* were “BSASTC”, “YASCGB”, and “CAAVTS” (Figures S4 and S5). The corresponding PWMs are provided (Tables S3 and S4). The enrichment of G/C at positions immediately adjacent to 6mA suggests that 6mA exhibits sequence preference. We did not detect enrichment of the “AGG” motif that is widespread in *C. elegans* (Greer et al. 2015), *A. thaliana* (Liang et al. 2018), and humans (Xiao et al. 2018). However, we did observe enrichment of similar motifs such as “ASG” and “AYG”, which resemble the “AGG” motif. The motif sequence “CCAWTCH”, “CTANTC” and “AHATWY” show pronounced AT enrichment. These motifs are comparable to the AT‐rich 6mA‐associated motifs reported in *Chlamydomonas* (Fu et al. 2015). The identification of these motifs in the *B. lactea* genome provides strong support for the presence of 6mA modification in this species.

### 3.2 6mA Is Associated With Gene Expression

**FIGURE 3:**
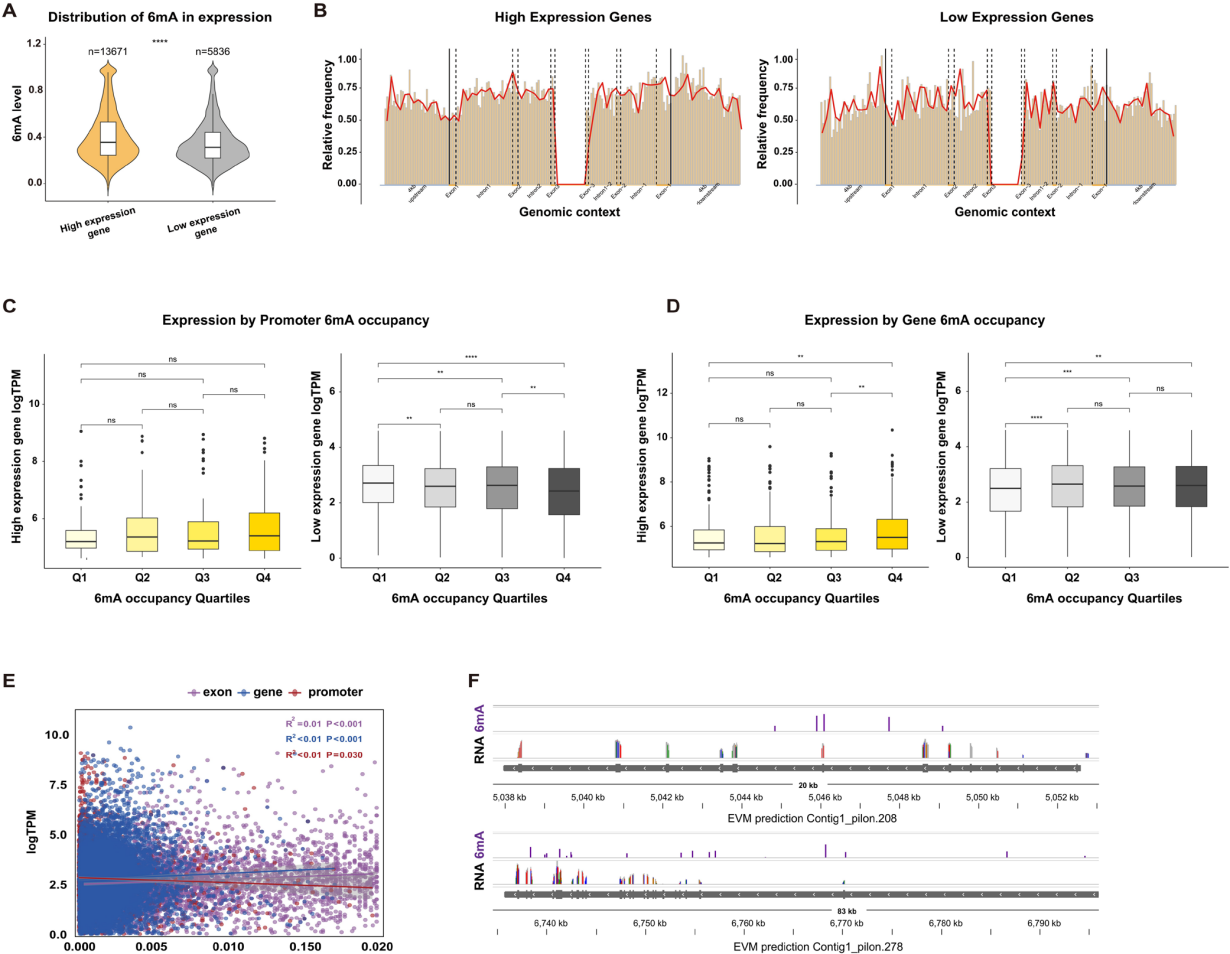
DNA 6mA is associated with gene transcription. (A) Two groups of genes with top 10% highest and bottom 10% lowest expression levels are plotted with 6mA levels. (B) Relative frequencies of 6mA sites across a normalized gene model. (C) The correlation between quartiles of 6mA occupancy in promoter regions and high expression level (left), low expression level (right). (D) The correlation between quartiles of 6mA occupancy in gene body regions and high expression level (left), low expression level (right). (E) There is a linear relationship between the 6mA occupancy in different sub‐regions of genes and expression level. (F) Genomic tracts showing two genes. In each panel, from top to bottom: 6mA level; RNA level; RefSeq gene track. ns, not significant; **p < 0.01; ****p < 0.0001.

Most recently, 6mA DNA methylation has been reported be involved in the regulation of gene expression in plants, animals, and humans (Liang et al. 2018; Wang et al. 2018; Xiao et al. 2018; Zhang et al. 2018; Shen et al. 2022; Yang et al. 2023). To understand the relationship between 6mA DNA methylation and gene expression in the *B. lactea* genome, we first categorized the genes into highly expression (TPM > Top 10%) and lowly expressed (TPM < Bottom 10%) groups. Our analysis revealed that genes with high expression levels exhibited significantly higher 6mA methylation levels than genes with low expression levels (Figure 3A). Consistently, an analysis of the relative frequency distribution of 6mA sites revealed an enrichment within the gene bodies (particularly exonic regions) of highly expressed genes, and a contrasting enrichment in the promoter regions of lowly expressed genes (Figure 3B). In line with this pattern, we found that higher 6mA occupancy in promoter regions was associated with lower gene expression, while having a minimal impact on the most highly expressed genes (Figure 3C). Conversely, higher 6mA occupancy in gene body regions was associated with higher gene expression (Figure 3D).

It is well known that high 6mA occupancy in exons is associated with active gene transcription in human (Xiao et al. 2018). Therefore, we further investigated the correlation between gene expression and 6mA methylation in different sub‐regions of genes. We found a weak positive correlation between 6mA occupancy in exonic regions (also in gene body regions) and gene expression level, and a weak negative correlation between 6mA occupancy in promoter regions and expression (Figure 3E), but no significant correlations in intronic regions (Figure S6). We also visualized the distribution patterns of 6mA and RNA‐seq read coverage across representative gene loci (Figure 3F). Thus, these findings indicated that 6mA in exonic regions is associated with active gene expression, while 6mA in promoter regions is linked to repressed gene expression in *B. lactea* .

### 3.3 Homeostatic Responses, Osmotic Equilibrium, and Metabolic Adjustments Facilitate Deep‐Sea Environmental Adaptation in *B. lactea*

**FIGURE 4:**
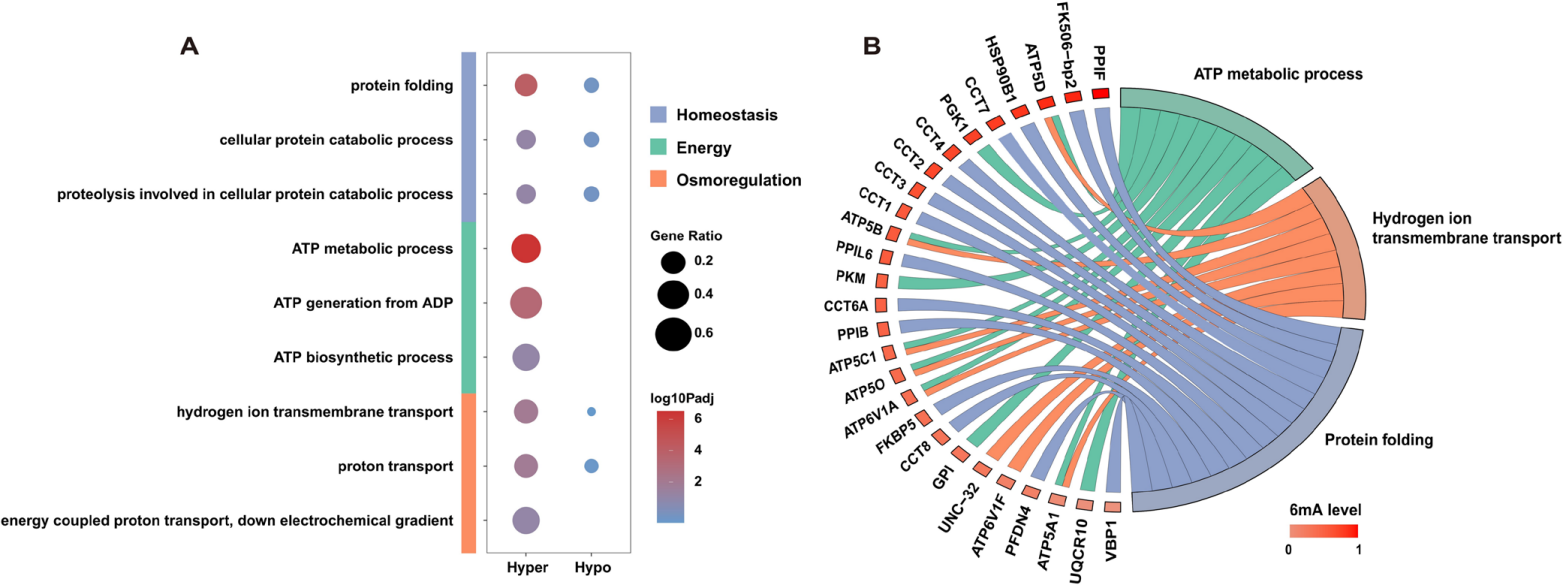
Functional enrichment analysis of 6mA‐methylated genes. (A) Gene Ontology (GO) biological process enrichment analysis depicting the interaction between hypermethylated‐highly expressed genes (top 10% gene body 6mA level; TPM > average) and hypomethylated‐highly expressed genes (bottom 10% gene body 6mA level; TPM > average). (B) Chord plot illustrating the relationship between genes and three GO terms. Each ribbon links a gene to a GO term, and gene methylation levels are indicated by a color scale.

To further explore the potential functions of 6mA, we performed GO annotation on all 21,122 predicted proteins of *B. lactea* using InterProScan (Jones et al. 2014), successfully annotating 10,715 proteins (50.1% annotation rate). We then performed GO enrichment analysis using comparing hypermethylated‐highly expressed genes (top 10% gene body 6mA level; TPM > average) (Table S5) against hypomethylated‐highly expressed genes (bottom 10% gene body 6mA level; TPM > average) as the control set (Table S6). Interestingly, we found that the hypermethylated‐highly expressed genes were predominantly associated with energy metabolism, protein homeostasis and osmotic equilibrium, particularly in ATP metabolic process, protein folding and hydrogen ion transmembrane transport (Figure 4A). These functional categories are generally associated with core cellular processes, including energy production, maintenance of protein stability, and regulation of intracellular ionic balance. In the present study, genes involved in protein folding exhibited higher 6mA methylation levels than genes in other functional categories, as shown by the chord plot (Figure 4B). To evaluate whether this filtering introduced bias, we had performed an additional GO enrichment analysis including all annotated genes, without applying an expression threshold. The results of this complementary analysis did not observe strong GO enrichment signals (adjusted *p* > 0.05; Tables S7 and S8). These results indicated that genes with high levels of gene body 6mA level show functional enrichment primarily within transcriptionally active genes.

### 3.4 Identification of Enzymes Involved in 6mA Methylation in *B. lactea*

Considering the abundant presence and potential importance of 6mA modification in the *B. lactea* genome, we sought to identify the candidate enzymes involved in 6mA methylation and demethylation. In *C. elegans* , DNA N6‐adenine methyltransferase 1 (DAMT‐1) was identified as a 6mA methylase through knock down and overexpression experiments (Greer et al. 2015). DAMT‐1 belongs to the MT‐A70 enzyme family, which includes AMT1‐AMT7 in *Tetrahymena* (Cheng et al. 2024; Wang et al. 2025), and METTL4 in silkworm and mammals (Wang et al. 2018). In contrast, N6‐methyladenine demethylase 1 (NMAD‐1), a member of the AlkB protein family, can directly catalyze 6mA demethylation in vivo, supporting its role as a 6mA demethylase in *C. elegans* . AlkB homologs have also been reported to function as 6mA demethylase in mouse, human, silkworms and rice (Yao et al. 2017; Wang et al. 2018; Xiao et al. 2018; Zhou et al. 2018; Zhang et al. 2020).

**FIGURE 5:**
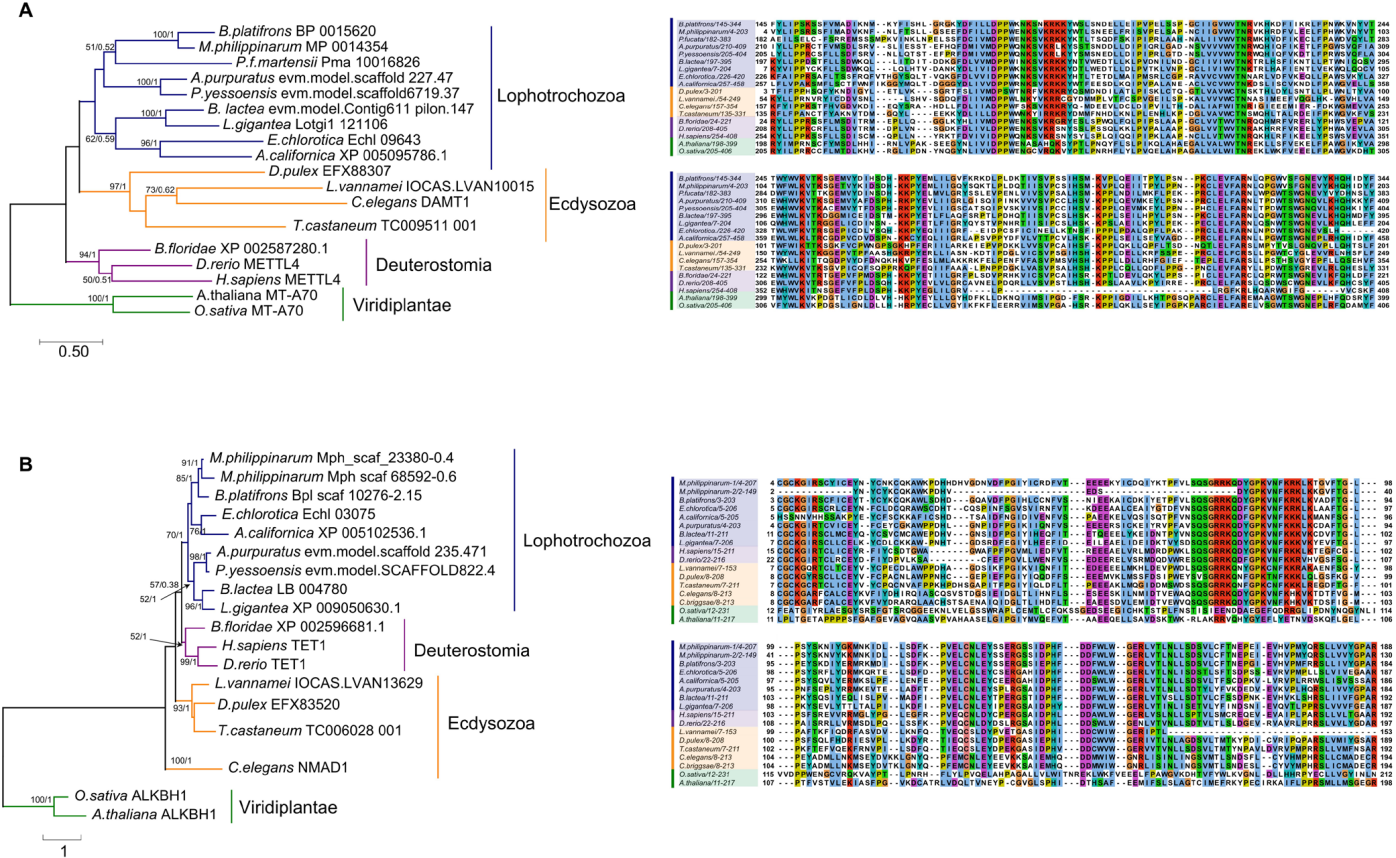
Discovery and identification of enzymes involved in 6mA methylation in *B. lactea* . Unrooted ML tree of the DAMT1 and NMAD1 family from 18 eukaryote species. Support values correspond to bootstrap values (1000 replicates) for ML tree and Bayesian posterior probabilities in this order; they are shown only for nodes with ≥ 50% support with two methods. (A) Phylogenetic tree and multiple protein sequence alignment of 6mA methyltransferase DAMT1. (B) Phylogenetic tree and multiple protein sequence alignment of 6mA demethylase NMAD.

We identified homologs of DAMT‐1 and NMAD‐1 in *B. lactea* , as well as in other representative species spanning eight lophotrochozoans ( *A. purpuratus* , *A. californica* , *B. platifrons*, *E. chlorotica* , *L. gigantea* , *M. philippinarum*, *P. yessoensis* , *P. fucata martensii*), four ecdysozoans ( *C. elegans* , *D. pulex* , *L. vannamei* , *T. castaneum* ), four ecdysozoans ( *C. elegans* , *D. pulex* , *L. vannamei* , *T. castaneum* ), three deuterostomes ( *B. floridae* , *D. rerio* , *H. sapiens* ), and two Viridiplantae ( *A. thaliana* , *O. sativa* ). Maximum‐likelihood phylogenetic analysis of the candidate DAMT and NMAD protein sequences revealed clear clades corresponding to these major lineages (Figure 5A). Multiple sequence alignment showed that the putative DAMT and NMAD proteins contain the conserved MT‐A70 and 2OG_FeII_Oxy_2 (AlkB) domains, respectively, in all examined organisms (Figure 5B). These results suggest that DAMT is more evolutionarily conserved than NMAD.

## 4 Discussion

DNA 6mA methylation is predominantly a prokaryotic DNA modification, and recent studies have suggested that 6mA modification is also present in many eukaryotic genomes (Luo et al. 2015; Boulias and Greer 2022; Shen et al. 2022; Yang et al. 2023). However, prior to this study, 6mA had not been reported in any lophotrochozoans or deep‐sea animals, leaving its presence and function in these groups unknown. Here, we present the first genome‐wide characterization of DNA 6mA in a deep‐sea cold‐seep species ( *B. lactea* ) using SMRT sequencing. We found 281,772 6mA sites, accounting for ~0.13% of total adenines in *B. lactea* . We further demonstrated that 6mA modification is associated with gene expression levels in this species. Moreover, GO enrichment analysis of 6mA‐methylated genes revealed that DNA 6mA in *B. lactea* plays a vital role in processes related to energy metabolism and environmental adaptation. Finally, we identified potential 6mA methyltransferase and demethylase enzymes in *B. lactea* that likely regulate genomic 6mA levels.

Compared to the well‐established role of 5mC in transcriptional repression in metazoans, the genomic distribution, abundance, and functional significance of 6mA have remained elusive. Our results have shown that approximately half of the 6mA sites in *B. lactea* are located in protein‐coding genes, whereas in *D. melanogaster* and mammals 6mA sites are predominately located in young long interspersed nuclear element 1 (LINE‐1) retrotransposon elements (Zhang et al. 2015; Wu et al. 2016; He et al. 2019). Given the large proportion of 6mA in exons relative to their genome coverage, our data indicate that DNA 6mA is strongly enriched in exonic regions of *B. lactea* protein‐coding genes. Consistent with this, 6mA peaks have also been found mainly in exons in embryonic zebrafish and the human genome (Liu et al. 2016; Xiao et al. 2018). In multi‐exon genes of *B. lactea* , introns are on average about 15 times longer than exons. Therefore, considering the relative lengths and counts of 6mA in different regions, we speculate that 6mA sites primarily function within exonic sequences in the *B. lactea* genome.

In this study, we also used the same batch of samples for RNA‐seq analysis. Comparative analysis of RNA‐seq and 6mA methylation in *B. lactea* uncovered that highly frequencies of 6mA modification in gene body regions correlate with higher gene expression. This positive relationship between 6mA occupancy and expression has also been observed in many other organisms, including *A. thaliana, C. reinhardtii, Tetrahymena*, rice, strawberry, human and fungi (Mondo et al. 2017; Liang et al. 2018; Xiao et al. 2018; Zhang et al. 2018; Xie et al. 2020; Fu et al. 2025; Wang et al. 2025). We further found that 6mA modification in promoter regions is associated with the repression of gene expression. This repressive role is consistent with previous observations in chicken and in transposable element genes of silkworms (Wang et al. 2018; Yang et al. 2023). These examples demonstrate the diversity of regulatory roles played by 6mA. Recently study on three deep‐sea worm species found that gene expression is positively correlated with gene body 5mC methylation and negatively correlated with promoter methylation (Perez et al. 2023). Our study identified 6mA methylation as a potentially important component of epigenetic regulation in this deep‐sea gastropod species, functioning analogously to 5mC in its context‐dependent influence on gene activity.

## 5 Conclusion

Overall, we have provided a genome‐wide 6mA distribution profile for the deep‐sea seep‐dwelling *B. lactea* . The distribution of 6mA in protein‐coding genes revealed that 6mA sites are enriched in exonic regions, but do not specifically target TEs. We further found that 6mA sites in the exonic regions are positively correlated with gene expression, and 6mA sites in the promoter regions are negatively correlated with gene expression, though to a lesser extent. The 6mA modifications of DNA, which appear to exert control on gene expression, could play a role in mechanisms of adaptation to deep‐sea environments. In particular, the hypermethylated‐highly expressed genes associated with homeostasis, osmoregulation, and energy metabolism suggest 6mA methylation contributes to physiological adaptation under extreme conditions. The epigenome of *B. lactea* would serve as a valuable reference for future investigations of the ecological roles of DNA 6mA modification in deep‐sea animals.

## Author Contributions

**Chengqin Chen:** data curation (equal), investigation (equal), writing – original draft (equal). **Jianhui Liu:** data curation (equal), methodology (equal). **Ruhao Zhuang:** data curation (equal), investigation (equal), methodology (equal). **Kuo Ni:** data curation (equal), methodology (equal). **Minxiao Wang:** data curation (equal), supervision (equal). **Yuli Li:** investigation (equal), writing – review and editing (equal). **Jing Wang:** investigation (equal), writing – review and editing (equal). **Shi Wang:** supervision (equal), writing – review and editing (equal). **Wentao Han:** data curation (equal), visualization (equal), writing – original draft (equal), writing – review and editing (equal). **Lisui Bao:** conceptualization (equal), funding acquisition (equal), project administration (equal), supervision (equal), writing – review and editing (equal).

## Disclosure

Benefit‐Sharing Statement: A research collaboration was developed with scientists providing genetic samples, all collaborators are included as co‐authors, the results of research have been shared with the provider communities and the broader scientific community. Benefits from this research accrue from the sharing of our data and results on public databases as described above.

## Conflicts of Interest

The authors declare no conflicts of interest.

## Supporting information


**Figure S1:** Bar graph shows the comparison between the numbers of observed 6 mA sites versus the expected sites in intron, exon, 5′ UTR and 3′ UTR regions.
**Figure S2:** Pie charts show the proportion of 6 mA sites distributed across promoters of 2 kb and 500 bp in length, as well as other genomic features.
**Figure S3:** Bar graph shows the comparison between the numbers of observed 6mA sites versus the expected sites in intron, exon, 5′ UTR and 3′ UTR regions.
**Figure S4:** The identified consensus motifs containing 6mA sites in genome.
**Figure S5:** The identified consensus motifs containing 6mA sites in exon regions.
**Figure S6:** The correlation between the methylation density on introns and expression level.

**Table S1:** Protein sequences for constructing a phylogenetic tree across the 18 species.
**Table S2:** Genomic Loci, fractions, and associated genes for 6mA sites in *Bathyacmaea lactea*.
**Table S3:** The PWMs of the identified consensus motifs containing 6mA sites in genome.
**Table S4:** The PWMs of the identified consensus motifs containing 6mA sites in exon regions.
**Table S5:** Gene ontology annotation for the hypermethylated‐highly expressed genes in *Bathyacmaea lactea*.
**Table S6:** Gene ontology annotation for the hypomethylated‐highly expressed genes in *Bathyacmaea lactea*.
**Table S7:** Gene ontology annotation for the hypermethylated genes in *Bathyacmaea lactea*.
**Table S8:** Gene ontology annotation for the hypomethylated genes in *Bathyacmaea lactea*.

## Data Availability

The PacBio sequencing data, short‐read RNA sequencing data, genome assembly and annotation files of *B. lactea* analyzed for this study can be found in NCBI (https://www.ncbi.nlm.nih.gov/bioproject/?term=PRJNA1237989) Sequence Read Archive (SRA) under BioProjectID PRJNA1237989. The raw sequencing reads are available under the accession number SRP57168351, and the final genome assembly under GCA_977020065.152. In addition, the genome assembly data and annotations have also been deposited at Figshare (https://doi.org/10.6084/m9.figshare.28928334). The Supporting Information have also been deposited at Figshare (https://doi.org/10.6084/m9.figshare.31640728).
