# Supplementary material for: Genome‐Wide 6mA Map Unveils Epigenetic Adaptation in Deep‐Sea Limpet
Source: Ecol Evol. 2026 Apr 8;16(4):e73449. doi: 10.1002/ece3.73449 (PMC13058721; doi:10.1002/ece3.73449)
Supplement: Supplementary file 1 — Figure S1: Bar graph shows the comparison between the numbers of observed 6 mA sites versus the expected sites in intron, exon, 5′ UTR and 3′ UTR regions. Figure S2: Pie charts show the proportion of 6 mA sites distributed across promoters of 2 kb and 500 bp in length, as well as other genomic features. Figure S3: Bar graph shows the comparison between the numbers of observed 6mA sites versus the expected sites in intron, exon, 5′ UTR and 3′ UTR regions. Figure S4: The identified consensus motifs containing 6mA sites in genome. Figure S5: The identified consensus motifs containing 6mA sites in exon regions. Figure S6: The correlation between the methylation density on introns and expression level. [file ECE3-16-e73449-s001.pdf]

Supplementary Materials

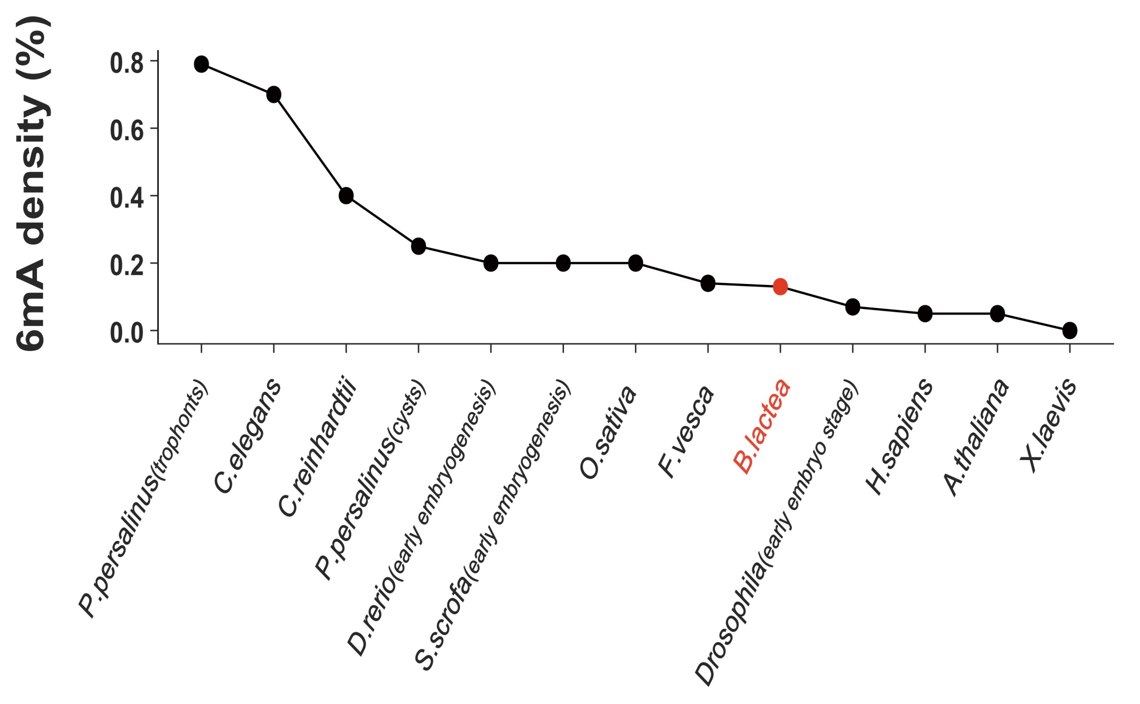

Supplementary Figure S1. Bar graph shows the comparison between the numbers of observed 6mA sites versus the expected sites in intron, exon, 5′ UTR and 3′ UTR regions.

Data sources: *Chlamydomonas reinhardtii* (Fu et al. 2015; Xiao et al. 2018), *Oryza sativa* (Zhou et al. 2018), *Caenorhabditis elegans* (Greer et al. 2015), *Drosophila melanogaster* (Zhang et al. 2015), *Homo sapiens* (Xiao et al. 2018; Tian et al., 2020), *Arabidopsis thaliana* (Liang et al. 2018), *Fragaria vesca* (Xie et al. 2019), *Danio rerio* (Liu et al. 2016), *Sus scrofa* (Liu et al. 2016), *Xenopus laevis* (Koziol et al. 2016), *Pseudocohnilembus persalinus* (Liu et al. 2024).

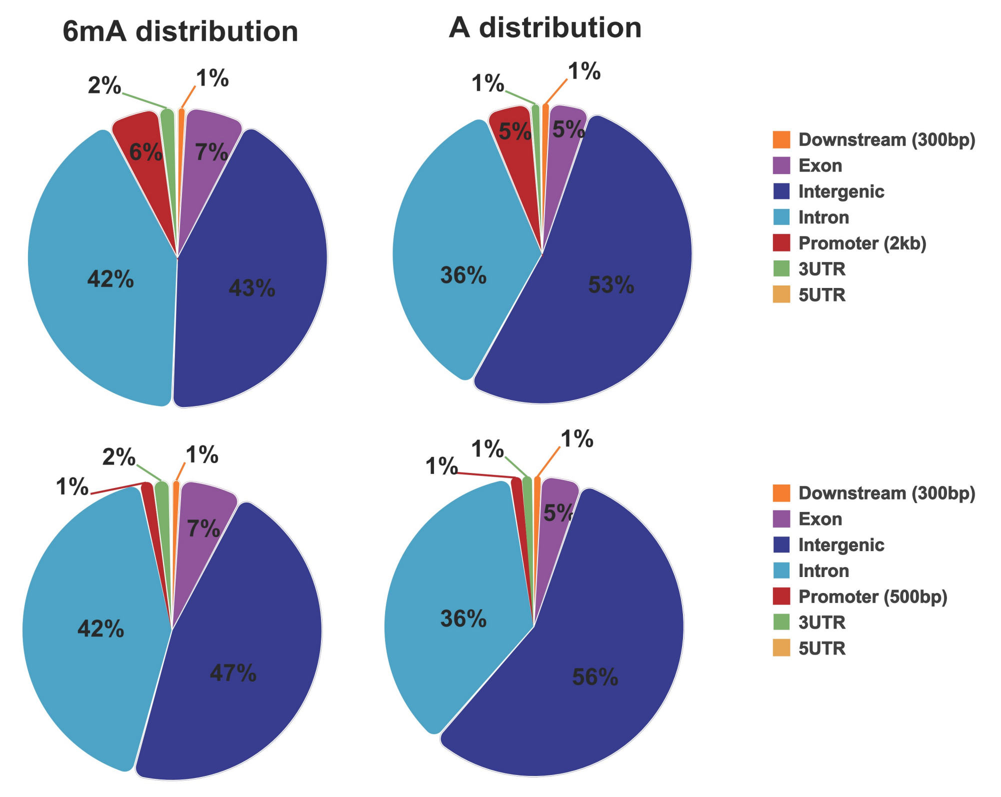

Supplementary Figure S2. Pie charts show the proportion of 6mA sites distributed across promoters of 2 kb and 500 bp in length, as well as other genomic features.

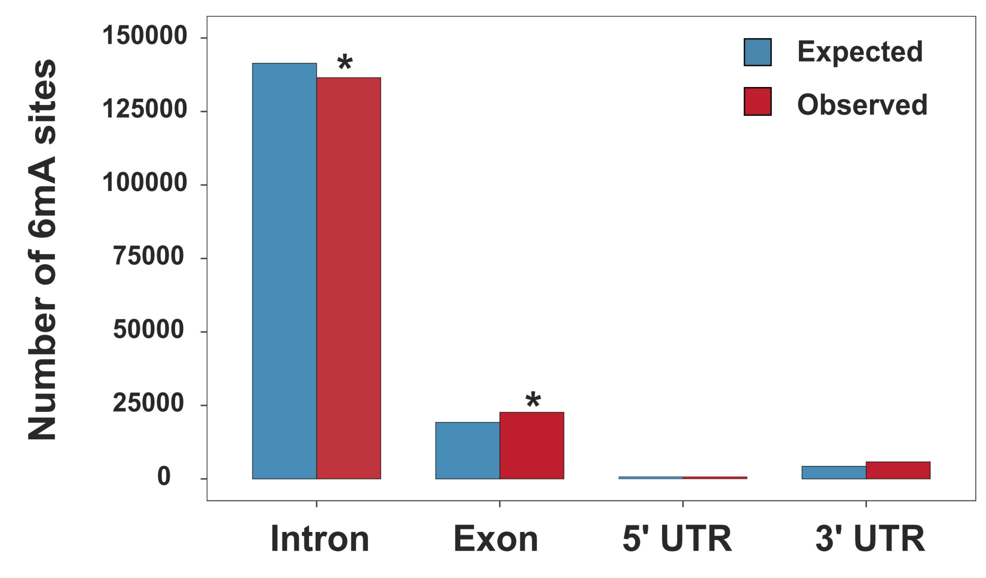

Supplementary Figure S3. Bar graph shows the comparison between the numbers of observed 6mA sites versus the expected sites in intron, exon, 5′ UTR and 3′ UTR regions.

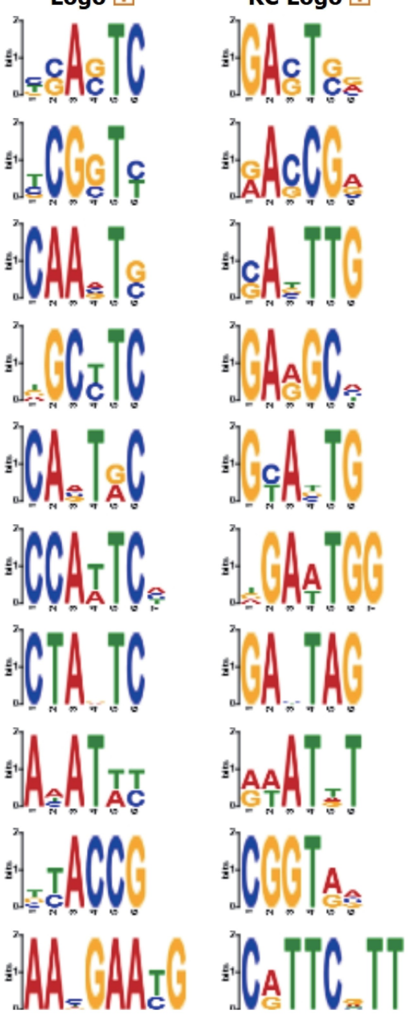

	Motif	Logo	RC Logo	E-value	Unerased E-value
1.	BSASTC			3.9e-1685	3.9e-1685
2.	BCGSTY			1.6e-1373	6.7e-1370
3.	CAAVTS			1.8e-497	1.8e-497
4.	DGCYTC			8.6e-343	2.7e-342
5.	CAVTRC			1.2e-266	3.9e-265
6.	CCAWTCH			1.2e-255	1.2e-255
7.	CTANTC			7.3e-254	1.3e-254
8.	AHATWY			1.0e-284	7.3e-303
9.	BYACCG			5.7e-209	7.9e-216
10.	AANGAAYG			1.0e-149	3.8e-245

Supplementary Figure S4. The identified consensus motifs containing 6mA sites in genome.

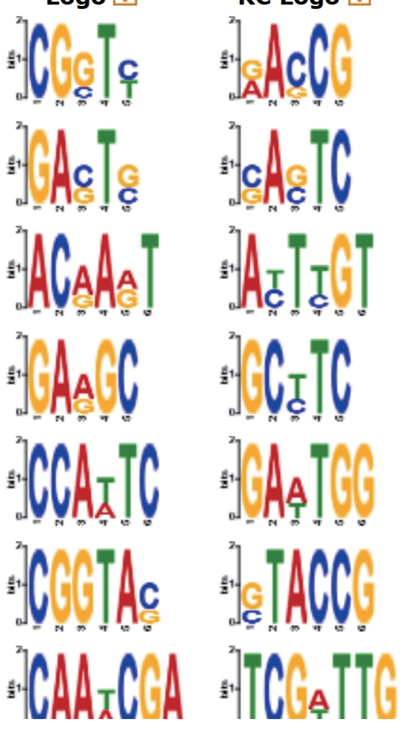

	Motif	Logo	RC Logo	E-value	Unerased E-value
1.	CGSTY			1.5e-024	1.5e-024
2.	GASTS			7.0e-020	7.0e-020
3.	ACRART			3.0e-005	4.0e-008
4.	GARGC			1.6e-004	7.2e-006
5.	CCAWTC			5.8e-004	2.4e-004
6.	CGGTAS			2.0e-003	2.0e-003
7.	CAAWCGA			2.2e-002	4.0e-003

Supplementary Figure S5. The identified consensus motifs containing 6mA sites in exon regions.

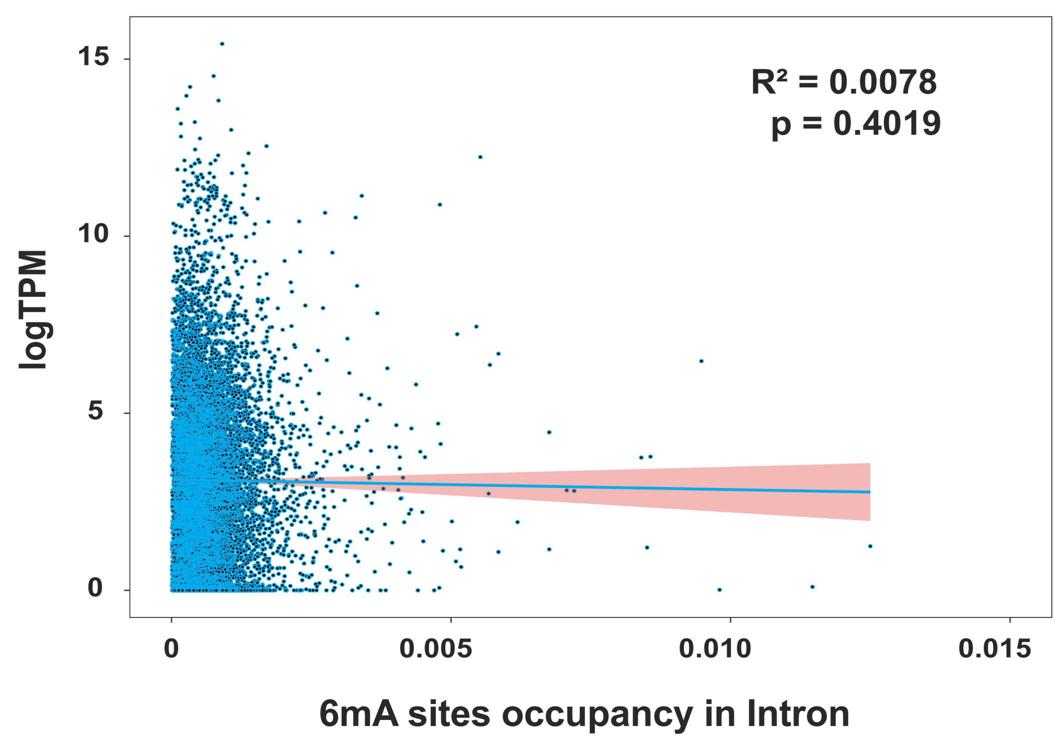

Supplementary Figure S6. The correlation between the methylation density on introns and expression level.

Supplementary Table S1. Protein sequences for constructing a phylogenetic tree across the 18 species.
Supplementary Table S2. Genomic Loci, fractions, and associated genes for 6mA sites in *Bathyacmaea lactea*.
Supplementary Table S3. The PWMs of the identified consensus motifs containing 6mA sites in genome.
Supplementary Table S4. The PWMs of the identified consensus motifs containing 6mA sites in exon regions.
Supplementary Table S5. Gene ontology annotation for the hypermethylated-highly expressed genes in *Bathyacmaea lactea*.
Supplementary Table S6. Gene ontology annotation for the hypomethylated-highly expressed genes in *Bathyacmaea lactea*.
Supplementary Table S7. Gene ontology annotation for the hypermethylated genes in *Bathyacmaea lactea*.
Supplementary Table S8. Gene ontology annotation for the hypomethylated genes in *Bathyacmaea lactea*.
