# Supplementary material for: Genome‐Wide 6mA Map Unveils Epigenetic Adaptation in Deep‐Sea Limpet
Source: Ecol Evol. 2026 Apr 8;16(4):e73449. doi: 10.1002/ece3.73449 (PMC13058721; doi:10.1002/ece3.73449)
Supplement: Supplementary file 2 — Table S1: Protein sequences for constructing a phylogenetic tree across the 18 species. Table S2: Genomic Loci, fractions, and associated genes for 6mA sites in Bathyacmaea lactea. Table S3: The PWMs of the identified consensus motifs containing 6mA sites in genome. Table S4: The PWMs of the identified consensus motifs containing 6mA sites in exon regions. Table S5: Gene ontology annotation for the hypermethylated‐highly expressed genes in Bathyacmaea lactea. Table S6: Gene ontology annotation for the hypomethylated‐highly expressed genes in Bathyacmaea lactea. Table S7: Gene ontology annotation for the hypermethylated genes in Bathyacmaea lactea. Table S8: Gene ontology annotation for the hypomethylated genes in Bathyacmaea lactea. [file ECE3-16-e73449-s002.zip › ECE3_73449__author.docx]

**Supplementary Table S1.** Protein sequences for constructing a phylogenetic tree across the 18 species.

**Supplementary Table S2.** Genomic Loci, fractions, and associated genes for 6mA sites in *Bathyacmaea lactea*.

**Supplementary Table S3.** The PWMs of the identified consensus motifs containing 6mA sites in genome.

**Supplementary Table S4.** The PWMs of the identified consensus motifs containing 6mA sites in exon regions.

**Supplementary Table S5.** Gene ontology annotation for the hypermethylated-highly expressed genes in *Bathyacmaea lactea*.

**Supplementary Table S6.** Gene ontology annotation for the hypomethylated-highly expressed genes in *Bathyacmaea lactea*.

**Supplementary Table S7.** Gene ontology annotation for the hypermethylated genes in *Bathyacmaea lactea*.

**Supplementary Table S8.** Gene ontology annotation for the hypomethylated genes in *Bathyacmaea lactea*.
